# Optimization of Ultrasound-Assisted Extraction Followed by Macroporous Resin Purification for Maximal Recovery of Functional Components and Removal of Toxic Components from* Ginkgo biloba* Leaves

**DOI:** 10.1155/2018/4598067

**Published:** 2018-09-12

**Authors:** Guisheng Zhou, Jiayan Ma, Yuping Tang, Xinmin Wang, Jing Zhang, Xin Yao, Wei Jiang, Jin-Ao Duan

**Affiliations:** ^1^Jiangsu Collaborative Innovation Center of Chinese Medicinal Resources Industrialization, Nanjing University of Chinese Medicine, Nanjing, 210023, China; ^2^College of Pharmacy and Shaanxi Collaborative Innovation Center of Chinese Medicinal Resources Industrialization, Shaanxi University of Chinese Medicine, Xi'an, 712046, China; ^3^Center for ADR Monitoring of Jiangsu, Nanjing, 210002, China; ^4^Department of Pharmacy, The First Affiliated Hospital of Soochow University, Suzhou, 215006, China; ^5^Taizhou Institute for Food and Drug Control, Taizhou, 225300, China

## Abstract

In the present study, the process of ultrasound-assisted extraction (UAE) followed by macroporous resin purification was successfully developed to achieve maximal recovery of functional components (flavonoids and ginkgolides) with minimal contents of toxic components (alkylphenols) from* Ginkgo biloba* leaves. Three effective extracted factors including HAc%, EtOH%, and UAE power were screened by Plackett–Burman design (PBD). The important variables were further optimized by rotatable central composite design (RCCD). By combination of PBD and RCCD, the resulting optimal UAE conditions were as follows: HAc% of 1.8%, EtOH% of 63%, ultrasound power of 303 W,* G. biloba* leaves powder amount of 1.0 g (solvent-to-solid ratio 40 mL/g), particle size of 60 mesh, extraction time of 40 min, and extraction temperature of 45°C. Under the optimum conditions, the yield of flavonoids was 25.1 ± 0.81 mg/g, ginkgolides was 10.6 ± 0.57 mg/g, and alkylphenols was 3.96 ± 0.31 mg/g. Moreover, the further enriching the functional components and removing toxic components from the obtained extracts of* G. biloba* leaves using the above optimum UAE condition was successfully achieved by macroporous resin DA-201. After column adsorption and desorption on DA-201, the content of total flavonoids was 36.51 ± 1.53%, ginkgolides was 13.24 ± 0.85%, and alkylphenols was 7.0 ± 1.0 *μ*g/g from the obtained dry extracts (drug to extract ratio of 45-50:1) of* G. biloba* leaves which were complied with Chinese pharmacopoeias.

## 1. Introduction

*Ginkgo biloba* (*G. biloba*) leaves have been commonly used to mitigate neurodegenerative diseases [1, 2], intermittent claudication [3], tinnitus [4, 5] and many other diseases [6]. Terpene lactones and flavonoids were considered to be the main active components for their beneficial effects [7]. Nowadays, the extract of* G. biloba* leaves (EGb) was standardized for the above two fractions of main functional components [7]. EGb has been of great interest to the food and pharmaceutical industries due to their inimitable benefits [8]. Therefore, an efficient process was required to achieve maximal recovery of natural terpene lactones and flavonoids from* G. biloba* leaves. However, the toxic constituents of alkylphenols were also detected in* G. biloba* leaves. It was reported that alkylphenols possessed allergenic, cytotoxic, embryotoxic, immunotoxic, mutagenic, and slight neurotoxic properties [7, 9]. Due to the negative properties of alkylphenols, a limiting concentration of 5 *μ*g/g for all alkylphenols was specified in the monographs for standardized EGb in the European and U.S. pharmacopoeias, while in the Chinese Pharmacopoeia the limiting concentration was 10 *μ*g/g [9]. Thus, for quality control and safety evaluation of drug or functional foods, efficient extraction and purification of EGb were of great importance to remove alkylphenols while retaining terpene lactones and flavonoids from* G. biloba* leaves. In the previous reports, some EGb were prepared in a multistep process to reduce alkylphenols while retaining terpene lactones and flavonoids from* G. biloba* leaves [7, 10]. Although it could enrich lactones and flavonoids while removing alkylphenols, the selective multistep process was complex in the practical operation and the optimal multistep process was proved to be expensive on an industrial scale.

A simple and cheap postprocess which could selectively remove alkylphenols while retaining terpene lactones and flavonoids from* G. biloba* product was proposed as an alternative solution to the above problem. Extraction and enrichment were often utilized as efficient and important techniques in the recovery and purification of target substances from plant materials.

Traditional extraction methods [7, 11, 12] including heating, boiling, Soxhlet, and accelerated solvent were developed for the recovery of terpene lactones and flavonoids from* G. biloba* leaves, but the content of alkylphenols was beside the point in these practices. Additionally, these methods were time-consuming, laborious, and inefficient. In recent years, a simple, efficient, and cheap extraction method of ultrasound-assisted extraction (UAE) was widely used for the extraction of effective components of plant materials [13]. From the previous reports, UAE exhibited the best mass transfer, cell disruption, solvent penetration, and capillary effect, and it did not damage the temperature sensitive botanical materials [14]. Moreover, UAE was the simplest and most economical technique and easy to scale up for industrial production [15]. In the process of UAE, many factors, including ultrasound power, time, temperature, and solvent-to-material ratio, could influence the extraction process, individually and collectively, and it was difficult to single out main independent variables to optimize. The conventional multivariable optimization was usually based on the “one-factor-at-a-time” approach, which was unable to detect interactions among independent variables and lack of complete information on effects of all determinants [16]. Recently, these limitations have been overcome by statistical experimental designs, which have emerged as powerful optimization tools. Plackett–Burman designs (PBDs) were frequently used for screening significant factors among multiple factors [17]. The significant factors were selected for further optimization, while factors exerting negligible effect on the response value were excluded from further experiments. Guided by the significant factors isolated by PBDs, researchers determined the relationships between variables and responses using the response surface method (RSM). PBDs combined with RSM (PBDs-RSM) was a useful method for evaluating multiple parameters and their interactions based on quantitative data and may effectively overcome the drawback of classic “one-factor-at-a-time” or “full-factors” approach [18, 19].

In the process of enrichment, macroporous resin has been widely used to adsorb and enrich target compounds in food chemistry and other fields owing to its high adsorption and desorption capacity, low cost, and simple equipment [20]. Furthermore, these resins were easily regenerated and not affected by the presence of inorganic materials [21]. Therefore, in this study, macroporous resin was employed to further enrich terpene lactones and flavonoids and remove alkylphenols in UAE solution.

The objectives of this study were to optimize a simple, safe, and low-cost process of UAE followed by macroporous resin purification to obtain maximum yield of flavonoids and terpene lactones with minimum alkylphenols content from* G. biloba* leaves. To the best of our knowledge, there was no report on PBDs-RSM application to the optimization of UAE conditions to obtain the high yield of terpene lactones and flavonoids with low content of alkylphenols in* G. biloba* leaves.

## 2. Experimental

### 2.1. Materials and Samples

**Figure 1:**
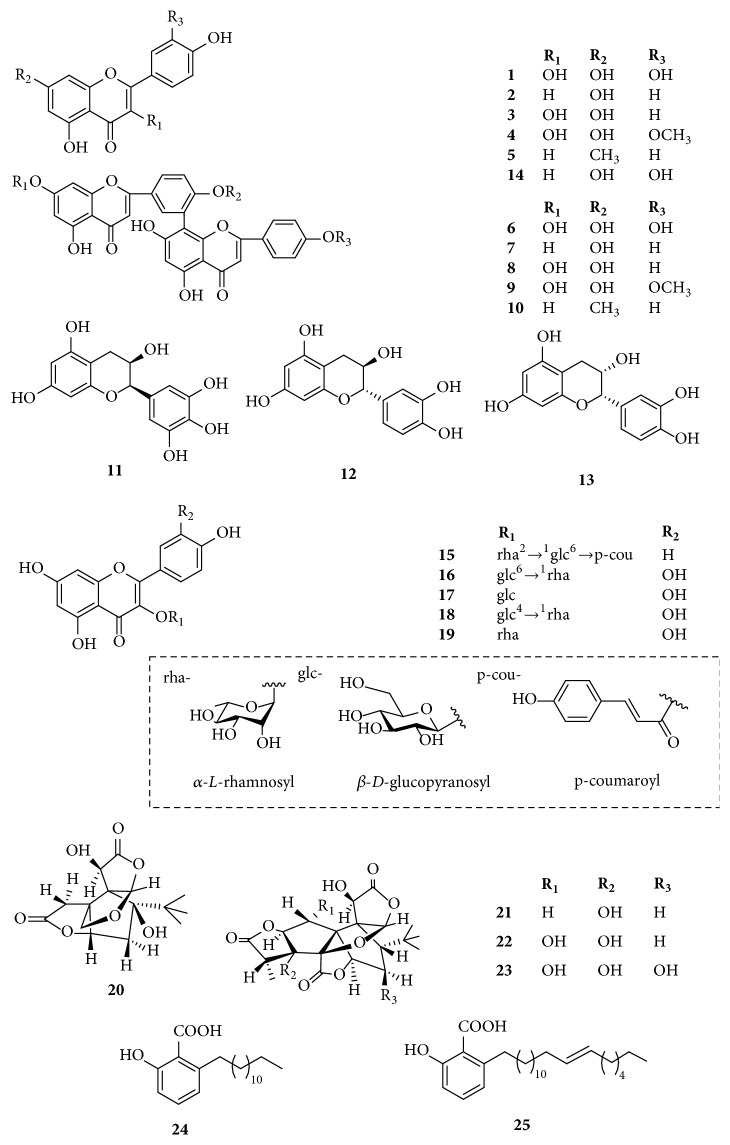
Chemical structures of the 25 investigated compounds. The code name of each compound represent the same meaning as described the sequence of each standard in Section 2.1.

The acetonitrile and formic acid were of HPLC grade. Deionized water was prepared from distilled water through a Milli–Q water purification system (Millipore, Bedford, MA, USA). DA-201, D101, D301, HP-20, HPD100, HPD400, AB-8, and S-8 were purchased from Cangzhou Baoen, Co. Ltd (Hebei, China). All other reagents were of analytical grade. The standards of quercetin (**1**), apigenin (**2**), kaempferol (**3**), isorhamnetin (**4**), genkwanin (**5**), amentoflavone (**6**), bilobetin (**7**), ginkgetin (**8**), isoginkgetin (**9**), sciadopitysin (**10**), (-)-epigallocatechin (**11**), (+)-catechin hydrate (**12**), (-)-epicatechin (**13**), luteolin (**14**), kaempferol 3-*O*-*α*-*L*-[6*‴*-p-coumaroyl-(*β*-*D*)- glucopyranosyl-(1,2)-rhamnopyranoside] (**15**), quercetin 3-*O*-[6-*O*-(*α*-*L*-rhamnosyl)- *β*-*D*-glucoside] (**16**), quercetin 3-*O*-*β*-*D*-glucoside (**17**), quercetin 3-*O*-[4-*O*- (*α*-L-rhamnosyl)-*β*-*D*-glucoside] (**18**), quercetin 3-*O*-*α*-L-rhamnoside (**19**), bilobalide (**20**), ginkgolide A (**21**), ginkgolide B (**22**), ginkgolide C (**23**), ginkgoneolic acid (**24**), and ginkgolic acid (**25**) were isolated from* G. biloba* in our laboratory and were identified by chemical and spectroscopic analysis [22]. The isolation protocols, yields, and spectroscopic data of these compounds from* G. biloba* were reported in the previous publications by our group [22–24]. The purity of each compound was > 98%. The structures of the 25 standards are shown in Figure 1.

The* G. biloba *leaves were collected from Taizhou, Jiangsu, China. A voucher specimen (NJTCU 20170708) was deposited at the Herbarium in Jiangsu Collaborative Innovation Center of Chinese Medicinal Resources Industrialization, Nanjing University of Chinese Medicine, Nanjing, China. After collection, the samples were air-dried.

### 2.2. Analytical Methods

In this study, UPLC–DAD–Q/TOF–MS was used to identify and determine terpene lactones, flavonoids, and alkylphenols. UPLC was performed using a Waters ACQUITY UPLC system (Waters, Milford, MA, USA), equipped with a binary solvent delivery system, an autosampler, and a photodiode array (PDA) detector. An Acquity BEH C_18_ column (2.1 mm × 100 mm, 1.7 *μ*m) maintained at 40°C was used with an injection volume of 2 *μ*L. Mobile phase A was 0.1% formic acid solution, and mobile phase B was acetonitrile solution. The flow rate was 0.4 mL/min. The linear gradient conditions were 95–80% A (0–1.5 min), 80–70% A (1.5–7.0 min), 70–35% A (7.0–9.5 min), 35–0% A (9.5–11.0 min), and 0% A (11.0–12.5 min). The wavelength of on-line PDA detector was set from 200 nm to 400 nm.

Mass spectrometry was carried out using a Waters Synapt mass spectrometer (Waters) equipped with an electrospray ionization source (ESI) in negative mode with scan range of* m/z* 50–1500 Da. The parameters in the source were set as follows: capillary voltage = 3 kV, cone voltage = 30 V, cone gas flow = 50 L/h, desolvation gas flow = 600 L/h, desolvation temperature = 350°C, and source temperature = 120°C. The accurate mass and elemental composition for the precursor and fragment ions were analyzed using the MassLynx V4.1 software (Waters).

### 2.3. Extraction Procedures

The extraction process was performed with an ultrasonic device (SY-5200T, Shanghai Shenyuan Ultrasonic Instrument Co. Shanghai, China). The extraction of* G. biloba *leaves was carried out using an ethanol (EtOH)/acetic acid (HAc)/water (H_2_O) solution at different ratio (%, v/v) and a total volume of 40.0 mL. Different amounts of* G. biloba *leaves powder and EtOH/HAc/H_2_O solution were mixed in each extraction (the factor of solvent-to-solid ratio) and sonicated at various times and temperatures under several sets of designed UAE conditions. After the UAE, the sample was centrifuged at 13000 rpm for 10 min to collect the supernatant for analysis.

### 2.4. Experimental Design of UAE Extraction Conditions

Experimental design was performed using a PBD followed with a rotatable central composite design (RCCD) in RSM. Initially, the PBD was applied to identify the significant variables that influenced the yields of terpene lactones, flavonoids, and alkylphenols from* G. biloba *leaves. Immediately, the variables of significance resulted from PBD were investigated by RCCD. PBD and RCCD were done by Design–Expert 8.5. The variables with confidence levels above 95% were considered as influencing the yield of target compounds from* G. biloba *leaves significantly.

#### 2.4.1. Plackett–Burman Design (PBD)

The PBD was used to screen out the multifactor and robust statistical significant factors that influence the applied procedure [25]. In this work, PBD was performed to evaluate the significance of seven variables which could affect the UAE procedures, including HAc%, EtOH%, solid amount, extraction time, UAE power, sample particle size, and extraction temperature. The design included 12 runs of various combinations of the independent variables coded as A–G and examined at high (+) or low (−) levels.  Table 1 shows the seven factors and their levels. The aim of this PBD was to identify the significant variables. The design matrix is presented in Table 2. The selected 7 parameters were realized with the yields of terpene lactones, flavonoids, and alkylphenols. The variables were screened out using a pareto chart. The chart displayed the absolute value of the effects and drew a reference line on the chart. All experiments were carried out in triplicate, and the mean value was taken as the response for PBD. PBD was based on the first-order polynomial model shown as standard equation

(1) $$\begin{array}{c} Y_{i}=C_{0}+\sum_{i}C_{i}X_{i} \end{array}$$

where *Y*_*i*_ is the experimental response, *X*_*i*_ is the independent variables, and *C*_0_ and *C*_*i*_ are the regression coefficients for intercept and linear terms, respectively.

#### 2.4.2. Rotatable Central Composite Design (RCCD)

To optimize the significant independent variables from PBD, a RCCD was applied to obtain maximum extraction of terpene lactones and flavonoids with minimum content of alkylphenols from* G. biloba* leaves. The significant independent variables were individually modified while the others were maintained at the 0 conditions. Each significant factor was examined at five levels (−*α*, −1, 0, 1, +*α*). The data from the RCCD were analyzed by multiple regressions to fit the following quadratic equation:

(2) $$\begin{array}{c} \Upsilon =\varphi_{0}+\overset{n}{\underset{i=1}{\sum }}\varphi_{i}x_{i}+\overset{n}{\underset{i=1}{\sum }}\varphi_{ii}x_{i}^{2}+\overset{n-1}{\underset{i=1}{\sum }}\overset{n}{\underset{j=i+1}{\sum }}\varphi_{ij}x_{i}x_{j} \end{array}$$

where *Υ*, *φ*_0_, *φ*_*i*_, *φ*_*ii*_, and *φ*_*ij*_ indicate the predicted response, the intercept term, the linear coefficient, the squared coefficient, and the interaction coefficient, respectively. Model adequacy was evaluated using F ratio and coefficient of determination (R^2^) represented at 5% level of significance, accordingly.

### 2.5. Experimental Design of Macroporous Resin Purification Conditions

The extract of* G. biloba* leaves was obtained by the above optimal UAE conditions. Then the extracts were concentrated to 4 mL under reduced pressure at 50°C and purified using a packed macroporous resin column. The column (300 mm × 18 mm I.D.) was packed with DA-201 macroporous resin. After loading of the sample (2 bed volume per hour, 2 BV/h), the column was washed with 3 BV (bed volume) of water and eluted with 6 BV of 80% ethanol aqueous solution successively at a flow rate of 2 BV/h. The effluent liquids were collected and analyzed by UPLC–DAD–Q/TOF–MS.

#### 2.5.1. Selection of Macroporous Resins by Static Adsorption and Desorption

Firstly, eight kinds of macroporous resins (DA-201, D101, D301, HP-20, HPD100, HPD400, AB-8, and S-8) were investigated for recovery of terpene lactones and flavonoids with minimum content of alkylphenols from extracts of* G. biloba* leaves by static adsorption. Adsorption experiments were carried out in 25 mL flasks in a water-bathing constant temperature vibrator (temperature was fixed at 40°C). Solid/liquid ratio (S/L) was set at 1:4, namely, 1 g of adsorbent (dry weight) for 4 mL of the above concentrated extracts. Desorption experiments were carried out in 50 mL flasks with the same operation as adsorption while S/L and temperature were, respectively, fixed at 1:20 and 40°C. The adsorption/desorption capacity and desorption ratio of each resin were calculated based on the following formulas (3), (4), and (5), respectively:

(3) Qa=C0−Ce×VeMr

(4) Qd=Cd×VdMr

(5) D=Cd×VdC0−Ce×Ve

where adsorption capacity represents the amount of target compounds (functional and toxic compounds from extracts of* G. biloba* leaves) adsorbed on 1.0 g dry resin; desorption capacity represents the amount of target compounds after desorption equilibrium; C_0_, C_e_, and C_d_ were the initial concentration, the equilibrium concentration (mg/mL) of target compounds in the extracts, and the concentration (mg/mL) of target compounds in desorption solution, respectively. Ve and V_d_ were the volume (mL) of the extracts and desorption solution (mL), respectively. M_r_ was the used amount of dry weight (g) of the resin.

#### 2.5.2. Selection of Macroporous Resins by Dynamic Adsorption and Desorption

According to the above result of static adsorption and desorption, DA-201 and AB-8 presented ideal adsorption and desorption capacities. Thus, DA-201 and AB-8 were further tested for the adsorption capacity of target compounds from* G. biloba* leaves extracts by dynamic adsorption at room temperature. The extracts (as described in Section 2.5) were filtered and loaded on a packed macroporous resin column with DA-201 and AB-8, respectively (2 BV/h). The effluent liquids were collected manually (1 BV for each) and analyzed by UPLC–DAD–Q/TOF–MS.

#### 2.5.3. Effect of Ethanol Concentration on Desorption of Target Compounds

The extracts (4 mL, as described in Section 2.5) were loaded on a glass column (300 mm × 18 mm I.D.) wet-packed with 10 mL of DA-201 (1 BV = 10 mL) at 2 BV/h. Then the same column was washed with 3 BV of water (2 BV/h) and eluted with 10%, 20%, 30%, 40%, 50%, 60%, 70%, 80%, 90%, and 100% ethanol aqueous solution (6 BV for each concentration gradient, 2 BV/h) successively. In sequence, the effluent liquid of different concentration gradient was collected and analyzed by UPLC–DAD–Q/TOF–MS.

#### 2.5.4. Effect of Elution Volume on Desorption of Target Compounds

From the result of Section 2.5.3, 80% ethanol aqueous solution was employed as elution solvent. 4 mL extracts were loaded on DA-201 column (2 BV/h). After washed with 3 BV of water, the column was successively eluted with 6 BV of 80% ethanol aqueous solution (2 BV/h). The effluent liquid was collected (1 BV for each) and analyzed by UPLC–DAD–Q/TOF–MS.

### 2.6. Statistical Treatment of Data

Design–Expert software (version 8.0, Stat-Ease, Minneapolis, USA) was used for designing experiments as well as for regression analysis of the experimental data obtained.

## 3. Results and Discussion

### 3.1. Identifying and Profiling of Functional and Toxic Components in G. biloba Leaves

It was necessary to develop a simple, rapid, and accurate analytical method to profile of flavonoids, terpene lactones, and alkylphenols in different matrices of* G. biloba* leaves. Based on several previously reported HPLC-DAD and HPLC-MS methods, we developed an UPLC–DAD–Q/TOF–MS method for the identification and determination of terpene lactones, flavonoids, and alkylphenols in* G. biloba* leaves. Using the developed UPLC–DAD–Q/TOF–MS, 25 components were unambiguously identified by comparing their retention times, UV spectra, the accurate masses, and fragment ions with those of respective standards. The characterization of compounds in* G. biloba* leaves was detailedly described in the previous reports [26–28].

**Figure 2:**
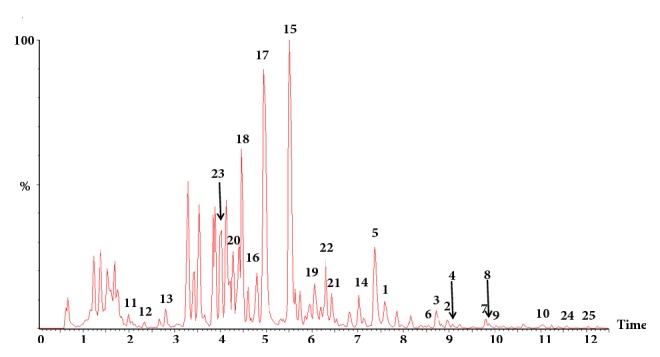
Representative chromatograms for the extract of* G. biloba* leaves monitored in negative mode with ESI-Q/TOF-MS. The peak numbers represent the same meanings as described the sequence of standards in Section 2.1.

For quantitative detection, an extract ion chromatogram (EIC) of the full scan MS experiment was validated and employed to assess the quantity of the 25 identified compounds in different matrices of* G. biloba* leaves. The extraction yield of the profile components was expressed as the total contents of flavonoids (TF, compounds** 1**-**19**) and ginkgolides (TG, compounds** 20**-**23**). The extraction yield of the toxic components was described as total contents of alkylphenols (TA, compounds** 24 **and** 25**). Representative chromatograms for the extract of* G. biloba* leaves are shown in Figure 2. The code numbers of compounds represent the same meanings as described the sequence of standards in Section 2.1.

*G. biloba* leaves were rich in flavonol glycosides, biflavones, and proanthocyanidins, and the flavonol glycosides were the most prevalent in* G. biloba* leaves and extracts. Numerous flavonol glycosides have been identified as derivatives of the aglycones quercetin, kaempferol, and isorhamnetin. Medicinal extracts made from dried ginkgo leaves were usually standardized to contain 24% flavonol glycosides which were considered to be related with their beneficial effects. The aglycones themselves occur only in relatively low concentration and thus were usually neglected for the determination of* G. biloba* extract as many literatures reported. For comprehensive evaluation of flavonols, UPLC–DAD–Q/TOF–MS was developed and used to simultaneously determine flavonol glycosides and aglycones from* G. biloba* extract in this study.

### 3.2. Screening of Significant Factors Using PBD

Two stages might be considered in method optimization: (a) a screening step, where many factors were studied to identify those with the significant effects on critical variables, and (b) the optimization, where the factors were further examined in order to determine the best conditions [16]. In the previous reports, the screening designs, such as the PBD, two-level full or fractional factorial designs, were the most widely used in the step of selection of factors because they were economic and efficient. Compared to two-level full or fractional factorial designs, PBD could identify main factors by a relatively few experiments. Therefore, PBD was selected as a method of screening design in this study.

**Figure 3:**
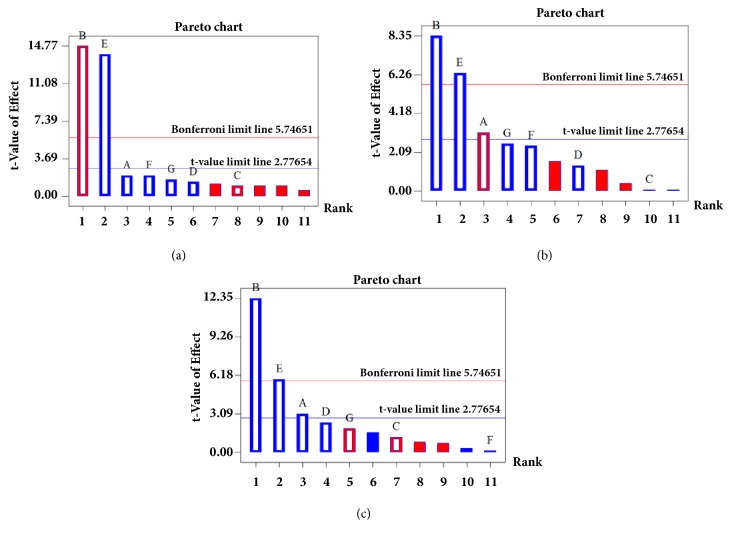
Pareto chart showing evaluated seven variables on the yields of flavonoids (a), ginkgolides (b), and alkylphenols (c) from* G. biloba* leaves. Variables with* t*-values higher than the critical value (2.776) were regarded as statistically significant.

Based on the preliminary tests and previous reports on the UAE, 7 parameters were determined as HAc% (A), EtOH% (B), solid amount (C), extraction time (D), UAE power (E), sample particle size (F), and extraction temperature (G) which were listed in Table 1. The experiment design matrix with Y_1_ (yield of TF), Y_2_ (yield of TG), and Y_3_ (yield of TA) as responses is also listed in Table 1, and the result and analysis of variance (ANOVA) are presented in Table 2. In general, variables of a large coefficient with a small* P*-value (<0.05) were considered as significant influence. Our results indicated that EtOH% (B) and UAE power (E) were the most effective parameters to the yield of TF (Y_1_), and HAc% (A), EtOH% (B), and UAE power (E) were considered as significant for responses (Y_2_ and Y_3_). From the previous reports, pareto chart could present the effect of factors on responses and check the statistical significance. In the pareto chart, two limit lines including Bonferroni limit line (5.746) and* t*-value limit line (2.776) were used to determine the extremely significant, significant, and insignificant coefficients of different factors when* t*-value of effect is above the Bonferroni limit line, between Bonferroni limit line and* t*-value limit line, and below* t*-value limit line, respectively [17]. Thus, the pareto chart could intuitively provide significant factors and it was employed to identify the significant factors in this study. The result of pareto chart plotted by the* t*-value of effect versus each parameter is shown in Figure 3. The* t*-value of EtOH% (B) and UAE power (E) was above Bonferroni limit line (shown in Figure 3(a)), which indicated that both EtOH% (B) and UAE power (E) were the extremely significant factors to the yield of TF (Y_1_). As shown in Figures 3(b) and 3(c), the* t*-value of EtOH% (B) and UAE power (E) was above Bonferroni limit line, and the* t*-value of HAc% (A) was between Bonferroni limit line and* t*-value limit line, both of which indicated that the three factors were considered as significant factors for the yields of TG (Y_2_) and TA (Y_3_). The R^2^ values of 99.07%, 98.61%, and 98.11% for yields of TF, TG, and TA indicated that the description of pareto charts was dependable. The initial first-order model equations developed by PBD for the yields of TF (Y_1_), TG (Y_2_), and TA (Y_3_) were generated by the Design–Expert 8.5 software according to

(6) Υ1=20.492+0.111A−0.08B−0.111C+0.006D+0.008E+0.017F+0.009G

(7) Υ2=−2.917−0.333A+0.082B−0.089C+0.007D+0.006E+0.028F+0.02G

(8) Υ3=−2.582+0.35A+0.139B−0.278C+0.02D+0.007E+0.002F−0.022G

Based on the final results of PBD, HAc% (A), EtOH% (B), and UAE power (E) were comprehensive considered as three important parameters for further UAE experiments of maximum yield of flavonoids and terpene lactones with minimum alkylphenols content from* G. biloba* leaves, whereas the rest 4 factors (solid amount, extraction time, sample particle size, and extraction temperature) contributed nonsignificantly. In this study, the effects of nonsignificant factors were also investigated in the preliminary experiments. The results indicated that a relatively low solid amount (namely high solvent-to-solid ratio) might help increase the contact chance between powders and extraction solvent, and raw materials with small particle size contributed to enlarge contact area between powders and extraction solvent. Therefore, fewer solid amount and smaller particle size might contribute to enhance the yield of target compounds. Similarly, the long extraction time may help increase the contact chance between raw materials powders and ultrasound wave or extraction solvent, thereby enhancing the yield of target compounds. Additionally, the appropriate rise of extraction temperature was advantage to the molecular motion, the penetration of solvent, and dissolution of target analytes. Considering the yield of functional and toxic target compounds, the conditions were selected as follows:* G. biloba* leaves powder amount of 1.0 g (solvent-to-solid ratio 40 mL/g), particle size of 60 mesh, extraction time of 40 min, and extraction temperature of 45°C. From the results, the high solvent-to-solid ratio (40 mL/g) was not feasible for industrial application, where low amounts of solvents were preferable. In this study, the results of high solvent-to-solid ratio were obtained from the small experiment of laboratory. Due to the lack conditions of industrial pilot magnification, 40 mL/g from the small experiment of laboratory was reported as the condition of solvent-to-solid ratio in the further research in this study.

### 3.3. Optimization of Significant Factors Using RCCD

The significant factors chosen from PBD* viz.* HAc% (A), EtOH% (B), and UAE power (E) were considered for further optimization using RCCD. The levels chosen for the factors were set based on the previous PBD analysis. The RCCD with *α* = 1.68 has been carried out on 20 experimental runs (2^3^+ (2 × 3) + 6), including 8 (2^3^) vertex points, 6 (2 × 3) axis points, and 6 center points. Multiple regression analysis was performed on the experimental data (Table 3) to evaluate for significance.

#### 3.3.1. Optimization of UAE Conditions for Maximum Extraction of Flavonoids

RCCD was applied to research the yield of flavonoids from* G. biloba* leaves using significant variables of A, B and E (Table 2). An initial response surface model of flavonoids yields from UAE was generated by the Design–Expert 8.5 software according to

(9) ΥTF=−70.86+0.86A+2.41B+0.12E−3.33AB+3.75AE+1.67BE−0.49A2−0.02B2−0.0002E2

where *Υ*_(TF)_, A, B, and E represent the predicted yield of flavonoids, the values of HAc%, EtOH%, and UAE power, respectively. ANOVA was then performed to retain the significant terms (*P *<0.05) and exclude the nonsignificant terms (*P *>0.05). This result indicated that E, B^2^, and E^2^ were significant. Consequently, a simplified model can thus be expressed as

(10) $$\begin{array}{c} \Upsilon_{\left(TF\right)}=-70.86+0.12E-0.02B^{2}-0.0002E^{2} \end{array}$$

The “*F*-value” of the model was 37.52, lack of fit value 3.73, and the value of “prob >* P*” < 0.0001, suggesting that it was an adequate model to accurately predict the response variable. The coefficient of determination (R^2^) was 0.9712 for the yield of flavonoids, indicating good agreement between experimental and predicted values. The predicted R^2^ of 0.82 was in reasonable agreement with adjusted R^2^ of 0.94.

**Figure 4:**
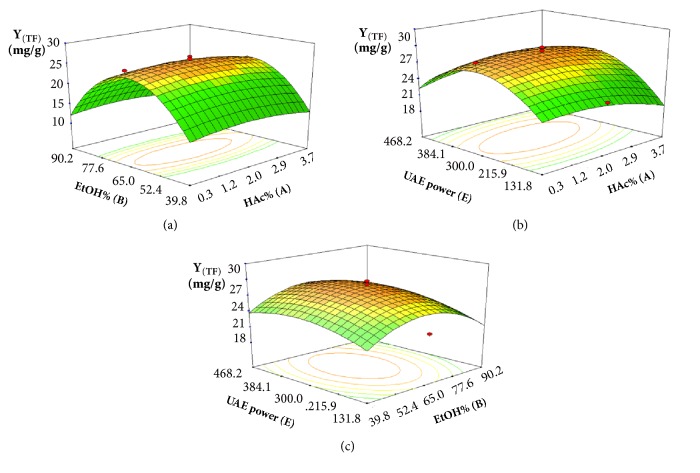
Three-dimensional contour plots showing the experimental factors and their mutual interactions: (a) effect of HAc% and EtOH% on the yield of flavonoids, (b) effect of HAc% and UAE power on the yield of flavonoids, and (c) effect of EtOH% and UAE power on the yield of flavonoids.

Three-dimensional response surfaces were developed by the fitted (10) and plotted by the response (Z-axis) according to two factors (X and Y coordinates), holding the other one factor at zero (0-level). Drawing RSM was regarded as the best way to visualize the influence of the independent variables. The value of *Υ*_(TF)_, EtOH% (B), and UAE power (E) contributed significant influences in both linear and quadratic manners, while the interactive effects of HAc% (A), EtOH% (B), and UAE power (E) presented nonsignificant effects. The interactions between HAc% (A) and EtOH% (B) were presented in Figure 4(a), keeping ultrasound UAE power (C) at 0-level. From Figure 4(a), when HAc% (A) was fixed, *Υ*_(TF)_ rapidly increased with the increase of EtOH% (B) until reaching a maximum and then rapidly decreased. However, HAc% (A) had less of an effect on changing the value of *Υ*_(TF)_. From Figure 4(b), the surface was relatively flat, and the effect of HAc% (A) on the yield of flavonoids from* G. biloba* leaves was not obvious at a given value of UAE power (E). When HAc% (A) was at a certain value, the yield of flavonoids also increased and then decreased.  Figure 4(c) shows the effects of EtOH% (B) and UAE power (E) on the yield of flavonoids from* G. biloba* leaves. When EtOH% (B) was fixed, the value of *Υ*_(TF)_ increased with the increase of UAE power (E) until reaching a maximum and then decreased. Similarly, EtOH% (B) caused an initial increase and then decrease in *Υ*_(TF)_. This result indicated that both EtOH% (B) and UAE power (E) were important variables for extracting flavonoids from* G. biloba* leaves.

The maximum yield of flavonoids from* G. biloba* leaves was calculated as 25.9 mg/g in the following optimum UAE conditions: HAc% of 1.9%, EtOH% of 64.2%, ultrasound power of 317.5 W,* G. biloba* leaves powder amount of 1.0 g (solvent-to-solid ratio 40 mL/g), particle size of 60 mesh, extraction time of 40 min, and extraction temperature of 45°C.

#### 3.3.2. Optimization of UAE Conditions for Maximum Extraction of Ginkgolides

ANOVA was also used to test the significance of the model of ginkgolides yields. The statistical significance of all the terms of the model was tested by the* F*-value and the* P*-value. As shown in Table 3, the* F*-value of 23.73 showed that the model was very important and the* P*-value of 0.1452 indicated that the “Lack of fit” was not significant. Thus, the regression equation was not lack of fit. The R^2^ value was 0.96, which indicated that 96% of the variations could be explained by the predicted model. The R^2^_adj_ value of 0.92 indicated the high degree of correlation between the observed and predicted values. In this model, A, E, A^2^, B^2^, and E^2^ were important terms for maximum extraction of ginkgolides from* G. biloba* leaves. Consequently, a simplified model can thus be expressed as

(11) ΥTG=−38.22+1.12B+0.05E−0.65A2−0.009B2−0.00009E2

where *Υ*_(TG)_, A, B, and E represent the predicted yield of ginkgolides, the values of HAc%, EtOH%, and UAE power, respectively.

**Figure 5:**
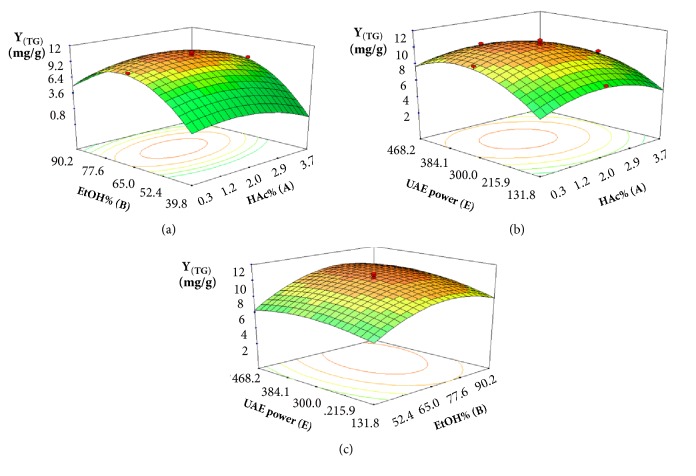
Three-dimensional contour plots showing the experimental factors and their mutual interactions: (a) effect of HAc% and EtOH% on the yield of ginkgolides, (b) effect of HAc% and UAE power on the yield of ginkgolides, and (c) effect of EtOH% and UAE power on the yield of ginkgolides.

Figure 5(a) shows the interaction of HAc% (A) and EtOH% (B), with a fixed UAE power (0 level). The linear and quadratic terms of HAc% (A) and EtOH% (B) caused significant influences on the yield of ginkgolides; however, the interactive effects between HAc% (A) and EtOH% (B) showed nonsignificant influences. As HAc% (A) increased, the value of Y_(TG)_ was increased slowly and then decreased slightly. The yield of ginkgolides was found to increase with the increase of EtOH% (B) and then level off at high EtOH% (B). The incomplete extraction may occur in a low EtOH% (B). However, higher EtOH% (B) contributed to improve dissolution of middle-polar compounds of ginkgolides, resulting in the continual enhancement on the yield of ginkgolides. The interactions between HAc% (A) and UAE power (E) are presented in Figure 5(b), keeping EtOH% (B) at 0 level. The significant effects on Y_(TG)_ were caused by both the linear and quadratic manners of HAc% (A) and UAE power (E), while the interactive effects between HAc% (A) and UAE power (E) exhibited nonsignificant influences on the yield of ginkgolides. When the HAc% (A) was fixed, the *Υ*_(TG)_ rapidly increased with the increase of UAE power (E) until reaching a maximum and then decreased slowly. However, HAc% (A) had less of an effect on changing the value of *Υ*_(TG)_. From the previous reports, more bubbles were formed and collapsed with larger amplitude ultrasound waves traveling through extraction solvent under high power in UAE process. As a result, violent shock wave and high-shear gradients might be created to cause the destruction of the cell walls. This facilitated the release of compounds significantly and enhanced the mass transfer rate simultaneously, thus leading to high yield of ginkgolides from* G. biloba* leaves. However, the degradation or isomerization of ginkgolides would occur under too high ultrasound power, which could explain the reason why the value of *Υ*_(TG)_ decreased.  Figure 5(c) presents the interaction of EtOH% (B) and UAE power (E), with a fixed liquid–solid ratio (0 level). EtOH% (B) and UAE power (E) contributed significant influences in both linear and quadratic manners, while the interactive effects between EtOH% (B) and UAE power (E) presented nonsignificant effects. When EtOH% (B) was fixed, the value *Υ*_(TG)_ increased with the increase of UAE power (E) until reaching a maximum and then decreased. Similarly, EtOH% (B) caused an initial increase and then decrease in *Υ*_(TG)_.

After optimization by the RCCD software, the optimum conditions predicted were HAc% of 1.8%, EtOH% of 70.3%, ultrasound power of 338.5 W,* G. biloba* leaves powder amount of 1.0 g (solvent-to-solid ratio 40 mL/g), particle size of 60 mesh, extraction time of 40 min, and extraction temperature of 45°C, respectively. Under the optimum conditions, the theoretical *Υ*_(TG)_ was found to be 10.65 mg/g.

#### 3.3.3. Optimization of UAE Conditions for Minimum Extraction of Alkylphenols

To simultaneously optimize three responses of flavonoids, ginkgolides, and alkylphenols, a RCCD (20 runs) was also used for the optimization of effective parameters on UAE for the minimum extraction of toxic components (alkylphenols) from* G. biloba* leaves. The experimental data show in Table 3. ANOVA was then performed to remove the insignificant terms (*P* >0.05), resulting in the following model:

(12) $$\begin{array}{c} \Upsilon_{\left(TA\right)}=32.94-0.85B-0.02E+1.17A^{2}+7.88B^{2} \end{array}$$

where *Υ*_(TA)_, A, B, and E represent the predicted yield of alkylphenols, the values of HAc%, EtOH%, and UAE power, respectively. The combination of independent variables, result, and analysis of variance (ANOVA) was also listed in Table 3. According to the ANOVA results, there was a good agreement between the predicted values and observed data points (R^2^ = 0.91 for the yield of alkylphenols). Herein, R^2^ value of 0.91 implied that 91% of the total variations for the extraction of alkylphenols were attributed to the independent variables such as HAc%, EtOH%, and UAE power. The fitness of the model was evaluated by the lack of fit test (*P* >0.05). The* P*-value of lack of fit for the yield of alkylphenols was found to be 0.1452, indicating the good fitness of the model (Table 3).

**Figure 6:**
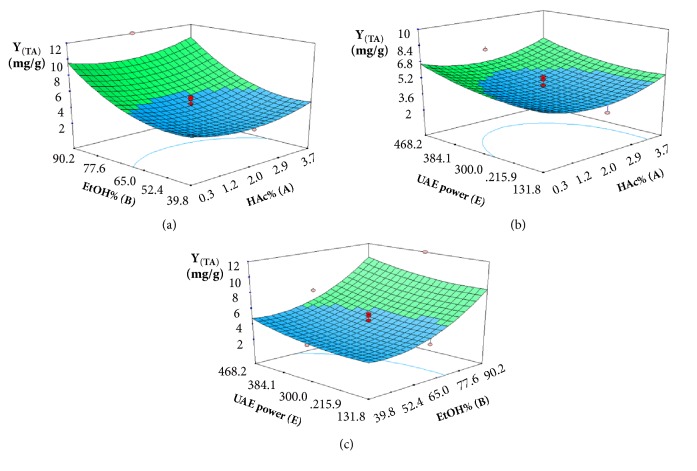
Three-dimensional contour plots showing the experimental factors and their mutual interactions: (a) effect of HAc% and EtOH% on the yield of alkylphenols, (b) effect of HAc% and UAE power on the yield of alkylphenols, and (c) effect of EtOH% and UAE power on the yield of alkylphenols.

The response surfaces were plotted (Figure 6) to investigate the interaction among the variables and to obtain the best UAE conditions of each variable for reaching the minimum extraction of alkylphenols from* G. biloba* leaves. The effect of HAc% (A) and EtOH% (B) was presented in Figure 6(a). The extraction yield of alkylphenols increased rapidly when HAc% (A) and EtOH% (B) were increased in the range of 1.9–3.7% and 53.7–90.2%, respectively. In other words, a greater removal of alkylphenols was obtained at lower HAc% (0.3–1.9%) and EtOH% (39.8-53.7%). From the previous reports, alkylphenols were anacardic acids and natural weak-polar compounds (6-alkylsalicylic acids). The above phenomenon could be explained by the fact that the suitable acidic condition was effective in increasing the hydrophobicity of alkylphenols, while more alkylphenols could be extracted into the solvent under higher percentage of EtOH. As shown in Figure 6(b), the extraction yield of alkylphenols increased rapidly when HAc% (A) and UAE power (E) were increased in the range of 1.9-3.7% and 228.5–468.2 W, respectively. On the contrary, extraction yield decreased slowly with increasing of HAc% from 0.3% to 1.9%, and extraction yield was also decreased slowly with increasing of UAE power (E) from 131.8 W to 228.5 W. The interaction relationship between EtOH% (B) and UAE power (E) on the yield of alkylphenols is shown in Figure 6(c). The yield of alkylphenols decreased with increasing EtOH% (B) from 39.8% to 53.7%. However, upon exceeding 53.7% of EtOH%, the yield of alkylphenols rapidly increased. Similarly, UAE power (E) caused an initial decrease and then increase in the yield of alkylphenols. This result confirmed that a lower yield of alkylphenols could be obtained at the low level of EtOH% (B) and ultrasonic power. Therefore, it was concluded that minimum yield of alkylphenols (the predicted value of 3 mg/g) could be achieved when HAc% was 1.9%, EtOH% was 53.7%, ultrasound power was 228.5 W,* G. biloba* leaves powder amount was 1.0 g (solvent-to-solid ratio 40 mL/g), particle size was 60 mesh, extraction time was 40 min, and extraction temperature was 45°C.

### 3.4. Optimization and Model Validation

In this study, considering the fact of maximum yield of functional components (flavonoids and ginkgolides) with minimum toxic components (alkylphenols) content, the Design–Expert 8.5 software was used for simultaneous optimization of the multiple responses by the desirability function. The desirability approach was a popular method that assigned a given score to responses and then a factor setting that maximizes and/or minimizes the overall score would be chosen. Using Design–Expert 8.5, the optimal values of significant factors for the yields of flavonoids, ginkgolides, and alkylphenols were provided as follows: HAc% of 1.8%, EtOH% of 63.3%, and ultrasound power of 302.7 W. Furthermore, the other nonsignificant factors including solid amount, extraction time, sample particle size, and extraction temperature were also investigated in the preliminary experiments and described in Section 3.2. Finally, for operational convenience, the following optimum conditions were selected: HAc% of 1.8%, EtOH% of 63%, ultrasound power of 303 W,* G. biloba* leaves powder amount of 1.0 g (solvent-to-solid ratio 40 mL/g), particle size of 60 mesh, extraction time of 40 min, and extraction temperature of 45°C which predicted the yields of flavonoids, ginkgolides, and alkylphenols as 25.8 mg/g, 10.2 mg/g, and 4.0 mg/g, respectively. Under the optimum conditions, the experimental yield of flavonoids was 25.1 ± 0.81 mg/g (*n* = 3), ginkgolides was 10.6 ± 0.57 mg/g (*n* = 3), and alkylphenols was 3.96 ± 0.31 mg/g (*n* = 3), which were close to the predicted values, indicating that the model was adequate for the extraction process.

### 3.5. Experimental Design of Macroporous Resin Purification Conditions

From the above experimental design of UAE conditions, the ideal UAE condition was obtained to achieve maximal recovery of functional components (flavonoids and ginkgolides) with minimum toxic components (alkylphenols) from* G. biloba* leaves, but the concentration of alkylphenols in extracts of* G. biloba* leaves using the optimized UAE condition still failed to comply with the standardized EGb in the European, US, and Chinese pharmacopoeias. To overcome this problem, macroporous resin was used to further enrich the functional components (flavonoids and ginkgolides) and remove toxic components (alkylphenols) from extracts of* G. biloba* leaves using the above optimized UAE condition.

#### 3.5.1. Selection of Macroporous Resins by Static Adsorption and Desorption

Adsorption and desorption process of macroporous resins on target constituents were not only influenced greatly by polarity and size of adsorbates, but also by dimensional structure of adsorbents, including specific surface area and pore diameter. Because weak-polar (alkylphenols), middle-polar (ginkgolides and flavonoid aglycones), and polar (flavonoid glycoside) compounds existed in the target analytes, thus the type of macroporous resins used in this research was selected among nonpolar, weak-polar, middle-polar, and polar compounds, which were applicable to the adsorption of the target compounds. Eight kinds of macroporous resins (DA-201, D101, D301, HP-20, HPD100, HPD400, AB-8, and S-8) were investigated and compared by the adsorption and desorption performance for flavonoids, ginkgolides, and alkylphenols. Compared to other resins including D101, D301, HP-20, HPD100, HPD400, and S-8, DA-201 and AB-8 presented adsorption and desorption capacities. Although the adsorption capacity of AB-8 was not as high as DA-201, desorption ratio of AB-8 was almost equal with DA-201. Thus, DA-201 and AB-8 were further investigated using dynamic adsorption experiment. The result of dynamic adsorption experiment indicated that the middle-polar (ginkgolides and flavonoid aglycones) and polar (flavonoid glycoside) compounds began to leak out obviously when 2 BV of the* G. biloba* leaves extracts were loaded on AB-8, while after 3 BV of the* G. biloba* extracts were loaded on DA-201 and the substances began to leak out apparently. Additionally, the weak-polar (alkylphenols) compounds began to leak out obviously when 4 BV of the* G. biloba* leaves extracts were loaded on AB-8, while after 6 BV of the extracts of* G. biloba* were loaded on DA-201, the alkylphenols began to leak out apparently. The more extracts of* G. biloba* were added, the more leak on AB-8 emerged than that on DA-201. Therefore, DA-201 macroporous resin was selected for further enrichment of the functional components (flavonoids and ginkgolides) and removed toxic components (alkylphenols) from the extracts of* G. biloba* leaves.

#### 3.5.2. Effect of Ethanol Concentration on Desorption of Target Compounds

**Figure 7:**
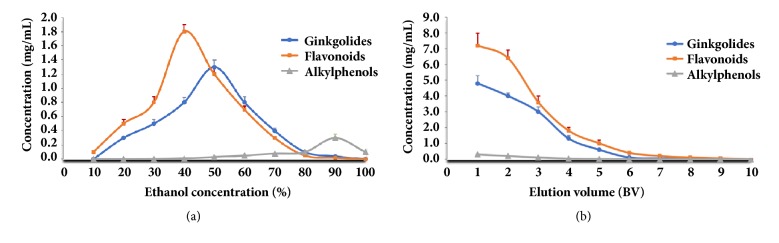
Elution conditions of flavonoids, ginkgolides, and alkylphenols from DA-201 macroporous resin. (a) Effect of ethanol concentration on desorption of total target compounds; (b) effect of elution volume on desorption of total target compounds. Results were expressed as the mean value ± standard deviation (*n* = 3).

To determine the proper ethanol concentration for desorption of target compounds from DA-201, continuous concentration gradient of ethanol aqueous solution was used to elute the adsorbed macroporous resin column successively. As shown in Figure 7(a), the more flavonoids could be cleared off when the higher the concentration of ethanol solution was employed as elution solution. More than 95% of flavonoids and ginkgolides were, respectively, eluted with 80% EtOH. From Figure 7(a), alkylphenols could not be eluted using 80% EtOH. Therefore, 80% EtOH was selected as elution solution for enriching the functional components and removing toxic components from the extracts of* G. biloba* leaves.

#### 3.5.3. Effect of Elution Volume on Desorption of Target Compounds

From Figure 7(b), the flavonoids and ginkgolides could be eluted partially with the first 4 BV of eluent when 80% EtOH was employed as elution solution. More than 95% of flavonoids and ginkgolides could be eluted when the eluent volume reached 6 BV. As shown in Figure 7(b), only few alkylphenols were eluted with 6 BV using 80% EtOH as the elution solution. Thus 6 BV was selected as elution volume in this study.

Finally, the purification process of target compounds on DA-201 resin was as follows: (1) adsorption stage: sample volume was 4 mL and flow rate was 2 BV/h, operated at room temperature; (2) washing stage: DA-201 resin was washed with 3 BV of water at a 2 BV/h flow rate; (3) desorption stage: elution solvent was 6 BV 80% EtOH at a flow rate of 2 BV/h.

### 3.6. Evaluation of the Validated Extraction Method

This overall study aimed at removing toxic components (alkylphenols) and retaining functional components (flavonoids and ginkgolides) from* G. biloba* leaves, which was highly desired from the perspective of food and pharmaceutical industries. Such extracts could be used as a good source of natural flavonoids and ginkgolides as nutraceuticals and pharmaceuticals. However, many of them with similarities in hydrophobicity increased the difficulties to separate functional components from alkylphenols. The UAE extraction followed by macroporous resin purification in this study could solve the above problem, and the method was simple, safe, and low cost. Under the optimized extraction and purification conditions, the content of total flavonoids was 36.51 ± 1.53% (*n* = 3), ginkgolides was 13.24 ± 0.85% (*n* = 3), and alkylphenols was 7.0 ± 1.0 *μ*g/g (*n* = 3) from the obtained dry extracts (drug to extract ratio of 45-50:1) of* G. biloba* leaves which met the standardized EGb in Chinese pharmacopoeias. The specification of alkylphenols, flavonoids, and ginkgolides of standardized EGb in Chinese Pharmacopoeia might be influenced by many factual factors including the quality of raw material (ginkgo leaves), technology of preparation, and condition of factory equipment. Thus, up to now, the Chinese Pharmacopoeia has specified alkylphenols (<10 *μ*g/g), flavonoids (>24%), and ginkgolides (>6%). However, the standardized EGb in Ph. Eur. and USP contained NLT (no less than) 22.0% and NMT (no more than) 27.0% of flavonoids, contained NLT 5.4% and NMT 12.0% of terpene lactones, and contained NMT 5 mg/kg of alkylphenols on the dried basis. The contents of total flavonoids and ginkgolides in the prepared extract of this study were, respectively, exceeded the NMT specifications of total flavonoids and ginkgolides in Ph. Eur. and USP. However, Chinese Pharmacopoeia only limited the NLT specifications of total flavonoids and ginkgolides from ginkgo extract but did not set the levels of NMT specifications. Thus, the prepared extract in this study met Chinese Pharmacopoeia and did not complied with Ph. Eur. and USP. Compared with Patent JP06/279300, the present method in this study possessed the practical value for its advantages of simple steps, easy operation, fast speed, and low cost. But the disadvantages of this study showed that the levels of target compounds did not meet Ph. Eur. and USP.

The drug-extract ratio (DER) was strictly speaking the “native” drug-extract ratio (DER native) declared in drug extracts. It specified the initial amount of drug used for the preparation of a certain amount of extract. This meant that various dried extracts of a drug can be compared in their quality and conclusions can be drawn as to the enrichment of the ingredients. In this study, the final DER was 45-50:1, which met the Chinese Pharmacopoeia, Ph. Eur. and/or USP.

## 4. Conclusion

In the present study, a simple and practical technology of UAE extraction combined with DA-201 macroporous resin purification was successfully optimized for maximum yield of functional components (flavonoids and ginkgolides) with minimum toxic components (alkylphenols) from extracts of* G. biloba* leaves. Under the optimal conditions, the specifications of alkylphenols, flavonoids, and ginkgolides of the obtained extract can reach the standard of EGb in Chinese pharmacopoeias. The obtained extract might be operational for the production of* G. biloba* leaves in Chinese food and pharmaceutical industries, though its specification of target components still did not meet the strict Ph. Eur. and USP.

## Figures and Tables

**Table 1:** Real values of the variables in the Plackett-Burman design and experimental data of the yields of flavonoids, ginkgolides. and alkylphenols from *G. biloba* leaves.

**Run**	**Factors**							**Responses**		
	**A** ^a^	**B**	**C**	**D**	**E**	**F**	**G**	**Y**_**1**_ ^b^	**Y**_**2**_	**Y**_**3**_
	(%)	(%)	(g)	(min)	(W)	(mesh)	(°C)	(mg/g)	(mg/g)	(mg/g)
1	0.0	80.0	0.5	50.0	400.0	40.0	60.0	18.5± 0.48	8.7± 0.53	11.5± 1.02
2	0.0	80.0	2.0	50.0	100.0	40.0	30.0	15.7± 0.52	6.2± 0.62	9.1± 0.52
3	0.0	50.0	2.0	10.0	400.0	60.0	30.0	20.4± 0.33	5.8± 0.35	6.5± 0.36
4	3.0	50.0	2.0	50.0	100.0	60.0	60.0	19.3± 0.41	4.8± 0.16	5.9± 0.49
5	0.0	50.0	0.5	50.0	100.0	60.0	60.0	18.9± 0.26	4.8± 0.22	4.3± 0.27
6	3.0	50.0	0.5	10.0	400.0	40.0	60.0	20.9± 0.29	5.1± 0.19	6.9± 0.19
7	3.0	80.0	2.0	10.0	100.0	40.0	60.0	16.2± 0.38	5.5± 0.42	8.5± 0.28
8	3.0	80.0	0.5	50.0	400.0	60.0	30.0	19.1± 0.23	8.1± 0.37	12.3± 0.82
9	0.0	50.0	0.5	10.0	100.0	40.0	30.0	18.5± 0.55	3.9± 0.28	4.5± 0.33
10	3.0	50.0	2.0	50.0	400.0	40.0	30.0	20.9± 0.61	4.4± 0.25	7.8± 0.26
11	0.0	80.0	2.0	10.0	400.0	60.0	60.0	18.7± 0.45	9.1± 0.31	9.4± 0.34
12	3.0	80.0	0.5	10.0	100.0	60.0	30.0	16.3± 0.17	5.3± 0.29	10.2± 0.92

^a^  **A**: HAc%; **B**: EtOH%; **C**: solid amount; **D**: extraction time; **E**: UAE power; **F**: sample particle size; **G**: extraction temperature.

^b^  **Y**_**1**_: the yield of flavonoids; **Y**_**2**_: the yield of ginkgolides; **Y**_**3**_: the yield of alkylphenols.

**Table 2:** Analysis of variance and regression analysis of Plackett-Burman design data for the prediction of significant extraction variables.

**Regression data**												
**Source**	**Y**_**1**_ ** (The yield of flavonoids)**				**Y**_**2**_ ** (The yield of ginkgolides)**				**Y**_**1**_ ** (The yield of alkylphenols)**			
	**Effect**	***F*-value**	***P*-value**	**Inference**	**Effect**	***F*-value**	***P*-value**	**Inference**	**Effect**	***F*-value**	***P*-value**	**Inference**
**Model**		61.03	0.0007	Significant		19.15	0.0063	Significant		29.64	0.0027	Significant
A	0.34	4.21	0.1094		0.88	9.86	0.0349	Significant	1.06	9.61	0.0362	Significant
B	2.40	218.27	0.0001	Significant	2.36	69.76	0.0011	Significant	4.18	152.54	0.0002	Significant
C	0.17	1.05	0.3629		0.016	0.004	0.9556		0.42	1.51	0.2860	
D	0.24	2.06	0.2242		0.38	1.86	0.2447		0.82	5.81	0.0735	
E	2.26	194.69	0.0002	Significant	1.78	40.17	0.0032	Significant	1.98	34.29	0.0042	Significant
F	0.34	4.21	0.1094		0.68	5.90	0.0721		0.05	0.022	0.8898	
G	0.26	2.69	0.1760		0.72	6.49	0.0635		0.66	3.68	0.1274	

**Table 3:** RCCD with experimental values for the yields of flavonoids, ginkgolides, and alkylphenols from *G. biloba* leaves, ANOVA for response surface quadratic model, and fit statistics for the response values.

**CCD experiments**						
**Run**	**A**	**B**	**E**	**Y**_**1**_	**Y**_**2**_	**Y**_**3**_
	**(**%**)**	**(**%**)**	**(W)**	**(mg/g)**	**(mg/g)**	**(mg/g)**

**1**	3.68	65.00	300.00	23.9±0.52	8.2±0.53	6.2±0.33
**2**	2.00	65.00	300.00	26.2±0.37	10.7±0.34	3.9±0.16
**3**	2.00	39.77	300.00	15.3±0.45	2.3±0.07	3.2±0.18
**4**	0.32	65.00	300.00	26.3±0.87	9.3±0.32	7.4±0.29
**5**	3.00	50.00	400.00	20.4±0.16	4.6±0.19	7.1±0.21
**6**	1.00	80.00	400.00	18.4±0.22	8.3±0.38	10.5±0.66
**7**	2.00	65.00	300.00	25.7±0.39	10.1±0.47	5.4±0.35
**8**	3.00	80.00	400.00	19.3±0.47	8.6±0.27	12.3±0.48
**9**	2.00	65.00	468.18	22.3±0.82	9.1±0.43	6.9±0.17
**10**	1.00	80.00	200.00	17.7±0.53	7.2±0.38	9.4±0.46
**11**	2.00	65.00	300.00	25.1±0.25	10.9±0.16	4.5±0.35
**12**	2.00	65.00	131.82	19.5±0.54	7.1±0.39	3.3±0.23
**13**	2.00	65.00	300.00	26.8±0.59	10.4±0.52	3.8±0.19
**14**	2.00	65.00	300.00	25.5±0.44	9.8±0.49	5.1±0.36
**15**	1.00	50.00	200.00	18.7±0.51	5.4±0.36	4.5±0.24
**16**	3.00	80.00	200.00	17.2±0.31	6.5±0.15	10.9±0.53
**17**	2.00	65.00	300.00	25.4±0.63	9.7±0.34	3.4±0.21
**18**	2.00	90.23	300.00	13.8±0.12	7.8±0.27	13.8±0.33
**19**	1.00	50.00	400.00	19.2±0.28	6.8±0.18	6.2±0.22
**20**	3.00	50.00	200.00	18.3±0.63	5.3±0.26	6.3±0.28

**Analysis of variance (ANOVA)**						

**Source**	**Y**_**1**_		**Y**_**2**_		**Y**_**3**_	
	***F*-value**	***P*-value**	***F*-value**	***P*-value**	***F*-value**	***P*-value**

Model	37.52	< 0.0001	23.73	< 0.0001	11.34	0.0004
A	0.65	0.4380	3.44	0.0933	0.69	0.4241
B	3.45	0.0929	52.37	< 0.0001	59.41	< 0.0001
E	8.29	0.0164	8.77	0.0143	5.35	0.0432
AB	0.022	0.8846	1.02	0.3354	0.027	0.8729
AE	1.25	0.2904	0.34	0.5709	0.027	0.8729
BE	0.06	0.9421	1.77	0.2125	0.000	1.0000
A^2^	3.89	0.0769	13.76	0.0040	11.85	0.0063
B^2^	284.79	< 0.0001	125.25	< 0.0001	27.12	0.0004
E^2^	62.49	< 0.0001	25.24	0.0005	2.82	0.1243
Lack of fit	3.73	0.0874	2.75	0.1452	4.40	0.0648

**Credibility analysis of the regression equations**						

**Index mark**	**SD**	**Mean**	**CV**%	**R** ^**2**^	**Adj.R** ^**2**^	**Pre. R** ^**2**^

**Y**_**1**_	0.95	21.25	4.47	0.97	0.94	0.82
**Y**_**2**_	0.66	7.90	8.40	0.96	0.92	0.71
**Y**_**3**_	1.29	6.71	19.28	0.91	0.83	0.42

## Data Availability

The data used to support the findings of this study are available from the corresponding author upon request.
